# Supplementary material for: Drug repurposing for aging research using model organisms
Source: Aging Cell. 2017 Jun 16;16(5):1006–15. doi: 10.1111/acel.12626 (PMC5595691; doi:10.1111/acel.12626)
Supplement: Supplementary file 7 — Data S1 Zip‐Archive of all report cards. [file ACEL-16-1006-s007.zip › RC_LYA.pdf]

LYA

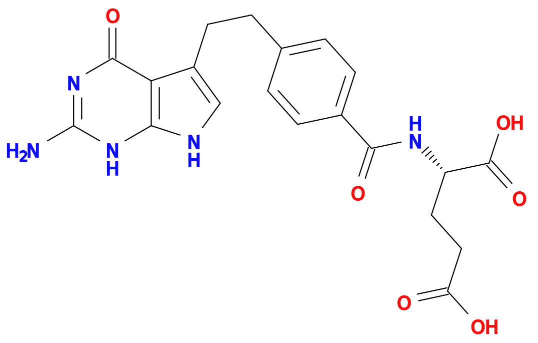

Database identifiers

ChEMBLCompound	CHEMBL225072
CHEBI	63616
eMolecules	30513153
eMolecules	37036813

Ranking

	Rank	Score
Drosophila	243/697	0.657
C. elegans	320/591	0.183

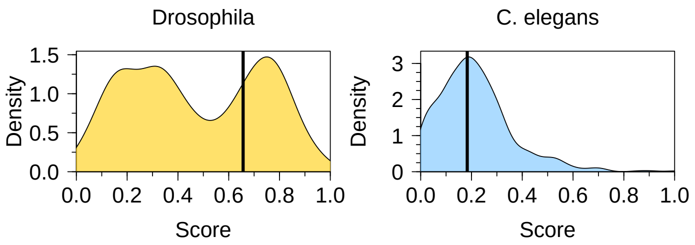

	Ageing implication	Domain conservation	Binding site conservation	Binding affinity	Bioavailability	Lipinski	Promiscuity	Purchasability	Drug approval	**Total**
Drosophila	0.792	0.933	1.0	0.913	(0.9)	-0.05	-0.0	0.1	0.0	0.657
C. elegans	0.792	0.935	0.983	0.913	0.201	-0.05	-0.0	0.1	0.0	0.183

Names

- **pemetrexed**
- (2S)-2-4-2-(2-amino-4-keto-1,7-dihydropyrrolo4,5-epyrimidin-5-yl)ethylbenzoylaminoglutaric acid
- (2S)-2-4-2-(2-amino-4-oxo-1,7-dihydropyrrolo4,5-epyrimidin-5-yl)ethylbenzoylaminopentanedioic acid
- (2S)-2-4-2-(2-amino-4-oxo-1,7-dihydropyrrolo4,5-epyrimidin-5-yl)ethylphenyl-oxomethylaminopentanedioic acid
- (2S)-2-4-2-(2-amino-4-oxo-1,7-dihydropyrrolo4,5-epyrimidin-5-yl)ethylphenylcarbonylaminopentanedioic acid
- 2-4-2-(2-amino-4-oxo-4,7-dihydro-3h-pyrrolo2,3-dpyrimidin-5-yl)-ethyl-benzoylamino-pentanedioic acid

Roles

antineoplastic agent, antimetabolite, EC 2.1.1.45 (thymidylate synthase) inhibitor, EC 1.5.1.3 (dihydrofolate reductase) inhibitor, EC 2.1.2.2 (phosphoribosylglycinamide formyltransferase) inhibitor

Status

Approved drug (according to ChEMBL)	No
Number of Rule of 5 violations	1
Binding affinity to original target in log units (RF-Score prediction)	7.35
Burns *C. elegans* bioavailability prediction	-10.22

Compound Target Characteristics

Thymidylate synthase

Best gene implication in ageing for this target family came from gene P45352 annotated in UniProt release 2014_02. Annotation GO 7568 (aging) was Inferred from Expression Pattern

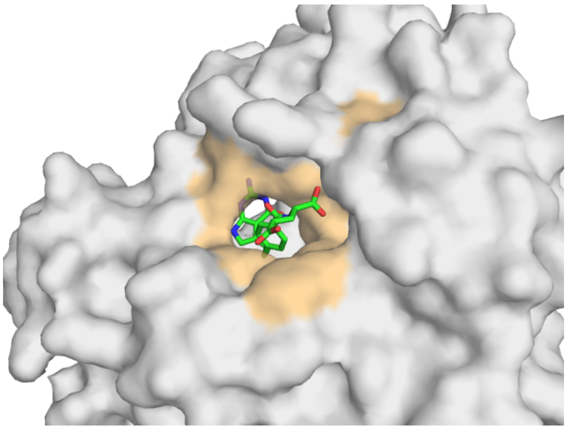

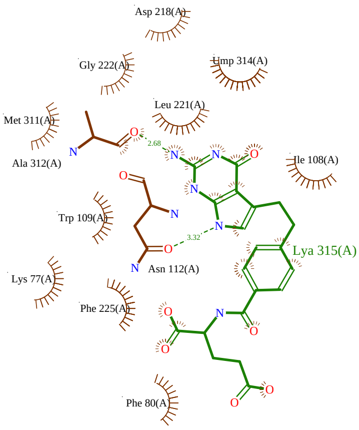

protein	amino acids contacts (binding site)
PDB:1ju6:chainA:P04818	K F I W N D L G F Y I M A
PDB:1juj:chainA:P04818	K F I W N D L G F Y I M A
sp:P04818:TYSY_HUMAN	K F I W N D L G F Y I M A
tr:Q53Y97:Q53Y97_HUMAN	K F I W N D L G F Y I M A
sp:P45352:TYSY_RAT	K F I W N D L G F Y I M A
sp:P07607:TYSY_MOUSE	K F I W N D L G F Y I M A
tr:Q544L2:Q544L2_MOUSE	K F I W N D L G F Y I M A
tr:Q541C9:Q541C9_DROME	K F I W N D L G F Y I M A
sp:O76511:TYSY_DROME	K F I W N D L G F Y I M A
tr:Q9Y052:Q9Y052_CAEEL	K Y I W N D L G F Y I M A
tr:Q9N588:Q9N588_CAEEL	K Y I W N D L G F Y I M A
sp:P06785:TYSY_YEAST	K F I W N D L G F Y I M S

	whole protein		domain-based		contact-based	
protein	ident	simil	ident	simil	ident	simil
PDB:1ju6:chainA:P04818	1.0	1.0	1.0	1.0	1.0	1.0
PDB:1juj:chainA:P04818	1.0	1.0	1.0	1.0	1.0	1.0
sp:P04818:TYSY_HUMAN	1.0	1.0	1.0	1.0	1.0	1.0
tr:Q53Y97:Q53Y97_HUMAN	1.0	1.0	1.0	1.0	1.0	1.0
sp:P45352:TYSY_RAT	0.87	0.95	0.92	0.98	1.0	1.0
sp:P07607:TYSY_MOUSE	0.88	0.95	0.92	0.98	1.0	1.0
tr:Q544L2:Q544L2_MOUSE	0.88	0.95	0.92	0.98	1.0	1.0
tr:Q541C9:Q541C9_DROME	0.62	0.84	0.65	0.86	1.0	1.0
sp:O76511:TYSY_DROME	0.62	0.84	0.65	0.86	1.0	1.0
tr:Q9Y052:Q9Y052_CAEEL	0.61	0.83	0.65	0.87	0.92	0.98
tr:Q9N588:Q9N588_CAEEL	0.6	0.83	0.65	0.87	0.92	0.98
sp:P06785:TYSY_YEAST	0.57	0.78	0.61	0.84	0.92	0.92

Ts (FBgn0024920) associated phenotypes

neuroanatomy defective

(Information from FlyBase)

Ts (UniProt:O76511) annotation
Pathway: Pyrimidine metabolism; dTTP biosynthesis.
Subunit: Homodimer.

(Information from UniProt)

WBGene00022455 associated phenotypes
lethal, sterile

(Information from WormBase)
